# Coenzyme Q10 and Cognition: A Review

**DOI:** 10.3390/nu17172896

**Published:** 2025-09-08

**Authors:** Madeleine C. Nankivell, Franklin Rosenfeldt, Andrew Pipingas, Matthew P. Pase, Jeffery M. Reddan, Con Stough

**Affiliations:** 1 Centre for Mental Health and Brain Sciences, Swinburne University, Melbourne, VIC 3122, Australia; 2 Baker Heart & Diabetes Institute, Melbourne, VIC 3004, Australia; 3 School of Psychological Sciences, Monash University, Melbourne, VIC 3168, Australia; 4 Metavate Consulting, Sydney, NSW 2000, Australia

**Keywords:** Coenzyme Q10, CoQ10, cognition, ageing

## Abstract

**Background and Objective:** With an increase in the number of older citizens in most Western countries, cognitive decline is becoming an increasingly significant issue. Numerous age-related metabolic and physiological changes, such as increased inflammation and oxidative stress, decreased adenosine triphosphate (ATP) production, poorer cardiovascular function, and reduced cerebral blood flow, have been implicated in cognitive decline, prompting research into interventions. Among these, Coenzyme Q10 (CoQ10), an antioxidant and metabolic stimulant, has shown promise in improving some of the underlying biological mechanisms of cognitive decline. However, not much is known about the efficacy of CoQ10 supplementation on cognition in the elderly. Therefore, the aim of this review is to explore the efficacy of CoQ10 supplementation on cognitive function. **Methods:** We conducted a review of animal studies and human clinical trials investigating the effect of CoQ10 supplementation on cognition in samples who were healthy or with specific diseases. Overall, twelve studies demonstrated improved cognitive function and two showed a reduction in oxidative stress in response to CoQ10 supplementation, either alone or in combination with other compounds. Out of eight human clinical trials in healthy subjects (n = 2) and disease states (n = 6), four showed evidence of a beneficial effect of CoQ10 supplementation on cognition, while two demonstrated an increase in cerebral blood flow. Disparity in the results of the clinical trials presented here is likely due to differing testing procedures, inconsistent use of cognitive assessments, and/or varying bioavailability of different preparations of CoQ10. **Conclusions:** There is some evidence to suggest that cognition and the biological mechanisms that regulate it are positively impacted by CoQ10 therapy. However, it is crucial to note that the literature presents mixed results, with many human clinical trials also reporting no benefit of CoQ10 supplementation on cognitive performance. To fully evaluate the benefits of CoQ10 on cognitive function in ageing and in neurodegenerative diseases, future studies are needed that target possible mechanisms and utilise a wider range of cognitive assessments.

## 1. Introduction

It is estimated that by 2050, 22% of the global population will be 60 years or older [1]. Associated with this changing demographic will be an increase in the number of people experiencing cognitive decline due to increasing age and prevalence of neurodegenerative diseases [2,3]. The causes of cognitive decline and neurodegenerative diseases are multifactorial but inflammation, oxidative stress, mitochondrial dysfunction, poorer cardiovascular health and reduced cerebral blood flow are major contributors [4,5,6,7]. These multiple processes occurring in the brain and body require different targeted therapeutic approaches.

A wide range of approaches have been used to counteract age-related cognitive decline, including dietary modification, physical exercise, and use of medication and dietary supplements [8,9]. While reviews of these approaches have often shown favorable outcomes, a comprehensive evaluation of the literature in areas such as the use of herbal supplements reveals a lack of high-quality evidence of efficacy, possibly due to inconsistencies in testing procedures [10]. Therefore, exploring new avenues is crucial, and CoQ10 supplementation with its unique biological effect on the body presents a promising option.

This review will provide an overview of CoQ10 and its physiological roles. It will summarise research on the beneficial effects of CoQ10 therapy in cardiovascular health, followed by a summary of studies demonstrating additional broader health benefits. Finally, a comprehensive literature review examining the effects of CoQ10 on cognition in both animal and human studies will be provided.

## 2. Understanding CoQ10: Evidence from Cardiovascular and Non-Cognitive Human Research

Coenzyme Q10 (CoQ10) is a naturally occurring fat-soluble antioxidant found in all cells in the body, mainly within mitochondria [11]. There are two main recognised forms of CoQ10, oxidised CoQ10 (ubiquinone), and the reduced form (ubiquinol). CoQ10 has inherently poor bioavailability, but various innovative formulations have been developed to enhance absorption [12]. Notably, ubiquinol (Ub) supplementation has high bioavailability and has been demonstrated to both improve in vivo CoQ10 absorption and clinical recovery in patients with severe heart failure [13,14]. Endogenous production of CoQ10 declines with age and is accompanied by an increase in reactive oxygen species (ROS) levels [11,15]. CoQ10’s primary function is within the mitochondrial electron transport chain, where it plays a crucial role in the synthesis of adenosine triphosphate (ATP) by transporting electrons from Complex I and Complex II to Complex III [16]. ATP is important for cellular function to maintain the health and energy of all bodily organs, especially those with high energy requirements such as the heart and brain.

### 2.1. CoQ10 Clinical Trials in the Cardiovascular System

CoQ10 supplementation has been shown to increase cellular energy production. In one clinical trial, patients scheduled for elective heart surgery were randomised to receive either oral CoQ10 (300 mg/d) or a placebo for two weeks prior to surgery. Analysis of myocardial tissue samples indicated that preoperative oral CoQ10 therapy elevated myocardial and cardiac mitochondrial CoQ10 levels and enhanced the efficiency of mitochondrial energy production [17].

A meta-analysis of published randomised placebo-controlled trials of CoQ10 therapy in heart failure (HF) up to 2013 revealed that CoQ10 supplementation markedly increased ejection fraction (EF) and improved the New York Heart Association (NYHA) functional class, a tool used to diagnose heart failure [12]. Specifically, supplementation led to a net enhancement of 3.67% in ejection fraction and a reduction of −0.30 in NYHA functional class [18].

In 2014, Mortensen et al. published a landmark prospective, randomised placebo-controlled multicenter trial of the efficacy of CoQ10 therapy in chronic heart failure (HF). Patients with moderate to severe heart failure (n = 420) were randomised to receive either CoQ10 at a dosage of 100 mg three times daily or a placebo, alongside normal care. The findings indicated that over the two-year follow up period, the CoQ10 group showed significant improvements relative to the placebo group in several key endpoints: cardiovascular mortality (9% vs. 16%, *p* = 0.026), all-cause mortality (10% vs. 18%, *p* = 0.018), and a reduced incidence of hospitalisation for heart failure (*p* = 0.033). Additionally, a notable improvement in NYHA class was observed in the CoQ10 group (*p* = 0.028). The conclusion was that long-term CoQ10 therapy in individuals with chronic heart failure is safe, alleviates symptoms, and reduces significant adverse cardiovascular events [19].

In a review published in 2007 of trials of CoQ10 in the management of hypertension, the overall efficacy and consistency of therapeutic action and incidence of side effects associated with CoQ10 treatment was assessed. Meta-analysis was performed using 12 clinical trials (362 patients) comprising three randomised controlled trials, one crossover study, and eight open label studies. The review concluded that CoQ10 has the potential to lower systolic blood pressure by up to 17 mm Hg and diastolic blood pressure by up to 10 mm Hg in hypertensive patients without significant side effects [20]. Subsequently, a meta-analysis by Zhao et al. containing 26 clinical trials (1831 participants) of CoQ10 in hypertension revealed decreased systolic blood pressure after CoQ10 supplementation in individuals with cardiometabolic diseases compared to control groups. The best effects were observed with CoQ10 dosages of 100–200 mg daily [21].

Research has shown that the bioavailability of CoQ10 within heart tissue and mitochondria has been found to significantly increase with CoQ10 supplementation [17]. In addition to strengthening the heart, CoQ10 also improves vasodilation in the circulatory system, supporting overall cardiovascular function [22]. Similarly, CoQ10 has been shown to improve blood flow in the upper limb in Type II diabetic patients [23]. A study by Kure et al. showed that, in patients with heart failure, patients with a higher bioavailability of CoQ10 displayed higher cerebral blood flow [24]. Moreover, given that reduced cerebral blood flow is one cause of impaired cognition in heart failure and in ageing, CoQ10 might have a beneficial effect on the brain and its function by increasing cerebral blood flow [24,25,26].

Whether CoQ10 can cross the blood–brain barrier (BBB) and have a direct effect on the brain is not clear. The BBB is created by the endothelial cells that line the blood vessels of the central nervous system. These cells possess unique properties which allow them to tightly regulate the movement of ions, molecules, and cells between the blood and the brain [27]. Recent work has identified many molecules required for proper BBB function as well as many of the cellular and molecular signalling events that regulate the formation of the BBB during development, its function in adulthood, and its response to injury and disease [28].

An in vitro study by Wainwright et al. using BBB endothelial cell models of CoQ10 deficiency, was the first to identify lipoprotein-associated uptake and efflux mechanisms regulating CoQ10 distribution across the BBB. The results implied that the uptake of exogenous CoQ10 into the brain might be improved by the administration of low-density lipoprotein receptor inhibitors or by interventions that stimulate luminal activity of SR-B1 (Scavenger Receptor) transporters [28].

Cardiovascular health is a known predictor of cognitive function. For example, a systematic review involving 50 studies, and over 100,000 participants (aged 18 to 100 years) determined that elevated blood pressures (including diagnosed hypertension) predicted poorer global cognitive functioning, memory, language, and attention (though more complex J- or U shaped associations were also apparent indicating too low blood pressures may also be detrimental) [29]. Likewise, elevated arterial stiffness is a pertinent predictor of reduced cognitive performance as reported in a systematic review by Alvarez-Bueno and colleagues [30]. Cerebral blood flow is also associated with cognitive function, with positive associations reported between cerebral blood flow and performance in different cognitive domains in the SABRE study [7]. Considering the varied benefits of CoQ10 supplementation on cardiovascular health outlined above, it is plausible that any benefits to cognition following CoQ10 supplementation may be, at least in part, due to positive effects upon cardiovascular health.

### 2.2. CoQ10 Clinical Trials in Other Systems

Evidence suggests that CoQ10 has a beneficial effect on physical exercise performance. In 41 participants, Cooke et al. administered CoQ10 for 14 days and observed an increase in plasma CoQ10 concentration, reduced oxidative stress, improved muscle blood flow, and improved exercise performance on a treadmill [31]. Siebrecht et al. reviewed 29 studies published between 1981 and 2012 on the effect of CoQ10 on physical exercise. They concluded that there are beneficial effects of CoQ10 on exercise performance and that the effect is more pronounced when the supplement is administered in its more bioavailable reduced form, ubiquinol, rather than in the oxidised form, ubiquinone. Additionally, greater benefits occur with higher doses (>300 mg/day) over a duration of two to three months [32].

Greater levels of physical activity have been reported to be positively associated with cognitive performance and reduced cognitive impairment [33,34,35] (e.g., Iso-Markku et al., 2024; Weuve et al., 2004; Zhu et al., 2018). Elevated physical activity likely benefits cognitive function via a range of mechanisms including benefits to cardiovascular health, but also inflammation (e.g., Kennedy et al., 2016) [36]. As CoQ10 supplementation may benefit physical performance, it is possible that CoQ10 supplementation could benefit cognition via improving one’s ability to engage in regular exercise, which would be a particularly important consideration in older adults.

Considering the rapid ageing of the general population, and older age being a pertinent predictor of neurodegenerative conditions such as dementia, the identification of potential interventions for supporting cognitive function is becoming increasingly important [37]. One such intervention may be supplementation with CoQ10. We have already provided a brief outline of some factors that appear to benefit from CoQ10 interventions (e.g., cardiovascular health, physical performance), which may also be pertinent predictors of cognitive function, especially in older adults. However, a more focused overview of the reported effects of CoQ10 administration upon cognitive performance is still required. Subsequently, the aim of this review is to examine the efficacy of CoQ10 supplementation on cognitive function in animal models and in human clinical trials.

## 3. Article Selection Method

To conduct this review, the authors searched for clinical trial papers using Scopus (n = 2249), PubMed (n = 288), and Cochrane library (n = 140) to search for clinical research conducted on the effects of CoQ10 on cognition. The complete list of key search terms were: (“CoQ10” or “Coenzyme Q10” or “Ubiquinol” or “coenzyme”) and (“cognition” or “cognitive decline” or “cognitive function” or “neurocognition” or “neuropsych*” or “memory”). All clinical trials were required to have measurable cognitive assessments. Overall, 12 relevant animal and in vitro studies and 8 human randomised controlled trials (RCTs) reporting cognitive outcomes were identified. These studies are outlined separately, with animal and in vitro research presented in one table and human clinical trials in another.

## 4. CoQ10 in Animal and In Vitro Cognitive Studies

Examining the mechanisms of CoQ10’s actions in animals assists in understanding the effects of CoQ10 on the brain and cognition. As such, Table 1 outlines the cognitive effects reported in the reviewed animal studies involving mice and rats, as well as details pertaining to potential mechanisms of effect (both in vitro and in vivo).

### 4.1. Ageing Mice

Like humans, mice exhibit a deterioration in cognitive functions as they age. Sumien et al. examined whether high or low dose CoQ10 supplementation, versus a normal diet, slowed age-related cognitive decline in mice. It was found that there was no significant effect of CoQ10 on the performance of mice completing the Morris water maze, measuring spatial memory and learning. CoQ10 levels were examined in the cortex, hippocampus-striatum, midbrain-diencephalon, cerebellum, and brainstem, with CoQ10 supplementation significantly increasing CoQ10 levels within the cortex [39]. These findings suggest that CoQ10 may have limited effects on cognitive function, despite its increased presence in brain tissue.

Building on the prior evidence supporting the benefits of co-administrating alpha-tocopherol (vitamin E) and CoQ10, Shetty et al. investigated their combined effect on cognition across multiple trials [38]. Mice were tested to assess if CoQ10, alpha-tocopherol, or both over three weeks could reduce age-related cognitive deficits. The results from the Morris water maze task showed no significant effect on performance due to any treatment [38]. Contrary to these findings, McDonald et al. demonstrated benefits of the combination of CoQ10 and alpha-tocopherol on learning. When completing a discriminated avoidance testing, the effect of CoQ10 and alpha-tocopherol given independently did not produce significant avoidance responses. However, mice taking the combination had significantly more avoidance responses in the learning phase, suggesting a greater ability to learn [40]. The varied outcomes of these trials indicate that additional research is necessary to fully comprehend the individual effects and interactions between these compounds.

### 4.2. Alzheimer’s Disease

Alzheimer’s disease (AD) is characterised by the accumulation of amyloid plaques and neurofibrillary tangles in the brain, with possible contributions from mitochondrial dysfunction and oxidative stress [52,53]. Ishrat et al. examined the impact of CoQ10 on cognitive deficits in a rat model of AD induced via the administration of streptozotocin, a diabetes-inducing chemical. CoQ10 therapy enhanced memory, learning and retention in rats with AD. Biochemical analysis indicated substantial reductions in oxidative stress and elevations in ATP levels inside the hippocampus and cerebral cortex of rats exhibiting AD. Rats undergoing CoQ10 therapy exhibited considerable enhancement in these metrics. The authors determined that CoQ10 treatment mitigated oxidative stress and enhanced ATP levels, thereby ameliorating learning and memory deficiencies [44].

The neuroprotective effects of CoQ10 were illustrated by Singh et al., including improved memory and learning in rats with cognitive dysfunction induced by amyloid beta injections into the hippocampal region. CoQ10’s beneficial impact was largely attributed to its ability to inhibit microglial activation, which plays a key role in the brain’s neuroinflammatory response. Furthermore, CoQ10 restored mitochondrial function. These biochemical and histopathological alterations highlight CoQ10’s potential as a therapeutic agent for AD, promoting improved cognitive outcomes [47].

The effects of CoQ10 on brain pathology and behaviour in genetically modified mice with AD were demonstrated by Dumont et al. Mice that were fed a diet supplemented with CoQ10 had lower carbonyl density, lower plaque numbers, and smaller plaque areas in their retrosplenial cortex and hippocampus. Amyloid beta levels were also lower in mice administered CoQ10 compared to the control group. CoQ10 administration also significantly decreased oxidative stress. CoQ10 treatment significantly enhanced cognitive performance in AD mice, as evidenced by improved learning compared to untreated controls. There were significant reductions in hippocampal plaque area and the number that significantly corresponded with improved spatial memory [42].

To better understand the mechanism of action of CoQ10 in improving cognition in AD, Ying et al. studied the relationship between CoQ10 and AD in genetically modified (3xTg-AD) AD mice. They found that administration of CoQ10 altered the expression levels of 12 proteins in the hippocampus of 3xTg-AD mice with a simultaneous improvement in spatial memory. Specifically, it was determined that there was an over-expression of CPLX-1 and CPLX-2 in AD mice following CoQ10 therapy. The authors proposed that this over-expression prevented spatial memory impairment [45].

The therapeutic potential of CoQ10 in AD was reported by Muthukumaran et al., demonstrating the efficacy of Co-Q10 in halting AD-related behavioral and pathological symptoms in a double transgenic mouse model. CoQ10 treatment significantly enhanced cognitive functions, including memory and learning, and appeared to prevent the progression of AD pathology by reducing oxidative stress, inflammation, and activating autophagy, particularly in the hippocampus. These findings suggest that if these results were translated to human studies, CoQ10 could improve the quality of life for individuals with neurodegenerative diseases, highlighting its role in therapeutic strategies [49].

Further evidence of CoQ10’s therapeutic potential was reported in a 2002 study by Sheykhhasan et al. This study in rats showed that CoQ10-loaded exosomes significantly enhanced cognitive function and memory in streptozotocin-induced AD. This was achieved by boosting brain-derived neurotrophic factor (BDNF) and SOX2 levels in the hippocampus, an area critical for cognitive function. Additionally, the treatment promoted neuronal differentiation, presenting a promising approach to AD therapy focused on neuroprotection and functional recovery [48].

In the brains of individuals with AD, poor neuronal survival has been associated with increased insulin resistance, which contributes to inflammation and the pathological changes observed [54]. Given this connection, supplements such as Biotin (vitamin B7) have been shown to positively influence insulin resistance in experimental diabetes [55]. Attia et al. investigated the neuroprotective effects of biotin and CoQ10 in an AD model induced by aluminium chloride in rats. The findings indicate that both biotin and CoQ10 independently, but more effectively when combined, can protect against AD by reducing neuroinflammation and enhancing insulin signalling within the brain, thus offering potential strategies for alleviating cognitive impairments and inflammation associated with AD [46].

Vitamins such as folic acid (vitamin B9) act as powerful antioxidants and are essential for cell synthesis, DNA transcription, and homocysteine remethylation—a process implicated in the development of AD [41,56]. The combination of folic acid and CoQ10 was investigated by Dolatabadi et al. to determine if it could mitigate the cognitive dysfunction in rats with lesion-induced AD. Rats with AD that received CoQ10 treatment showed significantly improved learning and memory in comparison to rats with AD who did not receive CoQ10 treatment [41]. More recently, rats with induced hypercholesterolemia (HC) and AD were studied by Fouad et al. to examine the effects of CoQ10 and Omega 3 fatty acids on cognition. The authors found that the CoQ10 and/or Omega 3 treated rats with AD were significantly faster than placebo rats when completing a maze, and experienced reductions in oxidative stress and an enhancement in cholinergic function. Additionally, rats with AD had histopathological evidence of brain deterioration; however, the combination treatment revealed nearly normal histological characteristics [43].

### 4.3. Epilepsy in Rats

Seizures resulting from heightened activation of brain neurons due to epilepsy are linked to neurodegeneration induced by reactive oxygen species (ROS) [57]. However, anticonvulsant drugs used for treating epilepsy, such as phenytoin, can additionally cause cognitive impairment and oxidative damage [58]. Research by Tawfik et al. aimed to investigate the effects of CoQ10 supplementation on oxidative stress, seizure severity, and cognitive function in epileptic rats. Two weeks of phenytoin impaired memory in rats, while CoQ10 supplementation improved it. Phenytoin administration significantly increased oxidative stress, but CoQ10 supplementation significantly ameliorated this increase [50].

### 4.4. Parkinson’s Disease

While many of the symptoms of Parkinson’s disease are motor-related, cognition, behaviour, and mood can also be negatively impacted [59]. In a preclinical investigation of CoQ10’s neuroprotective potential in Parkinson’s disease, Abu-Elfotuh et al. demonstrated that CoQ10 supplementation improved manganese-induced short-term memory impairments in a rat model. These effects were further enhanced when CoQ10 was combined with sesamol, thymol, and wheatgrass. Mechanistically, CoQ10 was associated with reductions in oxidative stress and neuroinflammation, as well as the modulation of apoptotic signalling pathways [51].

## 5. Human Clinical Research on Cognition

Insights gained from animal and in vitro studies have laid the groundwork for the development of human clinical trials. The final selection of human clinical trials investigating the effects of CoQ10 on cognitive function, identified through the literature search, is presented in Table 2.

### 5.1. Studies in Healthy Humans

Although CoQ10 supplements are widely available, research on their cognitive effect in healthy individuals is limited. To date, only two randomised clinical trials have been published. The first of these two trials was a randomised double-blind controlled trial by Kennedy et al. This study assessed the impact of a low dose CoQ10 (4.5 mg per day) in conjunction with multivitamin and mineral supplements in 106 females aged between 25 and 49 years. It was found that when completing cognitive tasks, cerebral blood flow significantly increased in the treatment group. However, there was no associated change in task performance. Although plasma CoQ10 serum levels exhibited a significant increase, it should be noted that the dosage of CoQ10 (4.5 mg/day) was probably too low to have an appreciable effect, and that there were 22 additional ingredients listed in the intervention used, many of which have been shown to have beneficial impacts on cerebral blood flow [60]. Considering this, the cerebral blood flow result may be attributable to the many components of the multivitamin and mineral supplement.

The second study in healthy humans was conducted by Kinoshita et al., using 50 mg Ub capsules twice daily, and revealed no statistically significant differences between groups in Memory Performance Index (MPI) score (memory), Trail Making Test (TMT) (attention and processing speed) times, and Digit Symbol Substitution Test (attention and processing speed) score [61]. Participants who were considered cognitively normal as per their MPI score at baseline demonstrated a significant improvement in memory after 34 weeks of Ub supplementation. Attention and processing speeds also improved as measured by the TMT. While these findings suggest a potential benefit of Ub in those who are cognitively normal, the study’s methodological shortcomings necessitate careful interpretation to consider their validity of these conclusions [61].

Specifically, statistical limitations may undermine the reliability and generalisability of the results, as the analyses conducted do not appear to fully substantiate the claims presented, underscoring the need for more rigorous statistical methodologies in future research. Additionally, the inclusion of participants with mild cognitive impairment (MCI) in the same dataset as cognitively normal individuals introduced potential inconsistencies, particularly since the classification criteria for MCI are not well explained. Notably, the selective exclusion of a participant at 34 weeks who was deemed to likely have dementia, which resulted in *p*-values narrowly meeting the conventional significance threshold. Further statistical issues include the absence of sample size calculation, an unverified assumption of normality in the data, no measures were taken to account for sex-based sampling bias, and the application of inappropriate analytical techniques. These limitations collectively weaken the credibility of the study’s conclusions and highlight the importance of more rigorous statistical methodologies in future research [61].

To further investigate the effects of CoQ10 on cognition in healthy humans, a randomised clinical trial was conducted by the current authors. This ongoing randomised controlled trial investigated the effects of Ub (200 mg/day) on cognitive decline over a 90-day period in healthy elderly participants aged 60 years or older. These participants were assessed and identified as experiencing subjective cognitive decline, thereby increasing the likelihood that they would benefit from the intervention. The primary outcome was cognition. Secondary outcomes included cardiovascular health, oxidative stress, liver function, and mood [68]. Results will be published in due course.

Given the paucity of studies in healthy individuals that help us understand whether Co-Q10 supplementation improves cognition, it is important to examine findings related to Co-Q10 supplementation and pathological conditions [51].

### 5.2. Alzheimer’s Disease

AD is an increasingly common disease especially in the elderly; however, there are few effective treatments for the disease [69]. After reviewing studies on the effects of CoQ10 and other antioxidants on AD, Galasko et al. identified which combinations of antioxidants are most effective and suggested their optimal dosages. Taking this information into consideration, the author’s aim was to investigate if these supplements would effect cognitive function and cerebrospinal fluid biomarkers of oxidative stress or neurodegeneration over 16 weeks. Three treatment groups were formed. The first group received a combination of vitamin C and E, along with α-lipoic acid, the second group received 400 mg of CoQ10 three times a day, while the third group received a matched placebo. However, CoQ10 showed no detectable effect on cerebrospinal fluid biomarkers such as tau, Aβ42, and F2-isoprostanes, suggesting it did not significantly impact oxidative stress or neurodegeneration. CoQ10 supplementation caused no significant changes in cognition [62].

### 5.3. Mild Cognitive Impairment

Mild cognitive impairment (MCI) is characterised by mild cognitive deficits with preserved daily functioning and is often considered a transitional phase between normal ageing and dementia. Importantly, amnestic MCI—marked primarily by memory impairment—is more strongly associated with progression to AD dementia, whereas non-amnestic MCI, which affects other cognitive domains, is more commonly linked to conditions such as vascular or frontotemporal dementia [63,70]. García-Carpintero et al. conducted a study with 69 patients with diagnosed MCI, using nine cognitive assessments for testing non-amnestic MCI and cerebral Doppler sonography. In addition, inflammatory markers in plasma were measured to assess the effects of 200 mg/day of Ub or placebo over the course of a year. The plasma concentration of Ub significantly increased after 1 year of CoQ10 supplementation. However, cerebral vasoreactivity improved and inflammation was reduced only in males. Neither sex demonstrated a change in cognition [63].

### 5.4. Parkinson’s Disease

Previous research has demonstrated that Ub significantly improves Parkinson’s disease symptoms, as assessed by the Unified Parkinson’s Disease Rating Scale [59]. While CoQ10 supplementation has been shown to improve mitochondrial function in Parkinson’s disease, randomised clinical trials combining CoQ10 with vitamin E found no improvement in self-reported cognitive function, despite its typical association with enhanced mitochondrial activity [71].

The same researchers investigated the tolerability of CoQ10 at doses up to 3000 mg/d in combination with 1200 IU/d of Vitamin E. CoQ10 plasma plateaued beyond 2400 mg/d, indicating that 2400 mg/d of CoQ10 and 1200 IU/d of Vitamin E represent the upper limit for effective dosing [72]. Considering this, Beal et al. sought to replicate this study using the same two dosage amounts which were found to be beneficial. No significant differences were seen between the cognitive function of those treated with CoQ10 and Vitamin E when compared to a placebo, demonstrating no cognitive clinical benefit [64].

Comparatively, the neuroprotective mechanisms of CoQ10 have been previously demonstrated when combined with creatine and given to Parkinson’s disease mice [73]. When pairing 100 mg CoQ10 three times a day and 5 g creatine twice a day together for Parkinson’s disease outpatients and inpatients, Li et al. found that cognitive function significantly improved when compared to the control group at 12 and 18 months. The comparison between the treatment group and control group revealed that the treatment group exhibited diminished phospholipid indicators, suggesting neuroprotective mechanisms, including enhanced mitochondrial activity and reduced oxidative stress [65].

### 5.5. Progressive Supranuclear Palsy

Progressive supranuclear palsy is categorised by symptoms such as akinesia/rigidity, postural instability, bulbar symptoms, speech/language dysfunction, ocular motor dysfunction, and frontal cognitive and behavioural dysfunction [74]. This condition is due to impairment in the respiratory chain as early as complex I (NADH dehydrogenase). This impairment reduces high-energy phosphate production due to a decrease in oxidative phosphorylation. CoQ10 supplementation, however, reduces neurotoxicity of complex I inhibitors [75]. It was for this reason that Stamelou et al. administered CoQ10 (5 mg/kg/d) to 21 progressive supranuclear palsy patients for 6 weeks. They reported a significant improvement in the frontal assessment battery (FAB) scores for the CoQ10 group compared to the control group, indicating an improvement in cognitive tasks involving the use of the frontal lobe [66]. This suggests that CoQ10 administration can restore the respiratory chain, and lead to the restoration of frontal lobe function.

### 5.6. Chronic Fatigue Syndrome

Chronic fatigue syndrome (CFS), also called myalgic encephalomyelitis (ME), is a poorly understood syndrome of unknown aetiology characterised by physical and mental fatigue made worse by physical and mental exertion and unexplained by any underlying medical condition [76]. The fatigue of CFS is accompanied by a range of additional medical symptoms including depression, impaired cognition and neurocognitive dysfunction [77,78]. A study by Fukuda et al. found that 150 mg of CoQ10 daily for both 8 and 12 weeks improved cognitive function in chronic fatigue patients. The treatment group showed improved performance in arithmetic tasks, working memory, and fatigue symptoms. Additionally, the CoQ10 supplementation group showed a decrease in oxidative stress levels, suggesting that reducing oxidative stress levels can improve the pathophysiology of chronic fatigue syndrome [67].

## 6. Discussion

In this review, multiple studies with animals have revealed protective effects of CoQ10 on cognition in healthy ageing and neurodegenerative disease models, though null effects have also been reported [38,62,64]. While human clinical trials have shown some evidence of benefit in disease states but none in healthy ageing, multiple studies reported null or non-significant results. While these null effects may be attributable, at least in part, to limitations such as small sample sizes and insufficient statistical power, rather than the absence of a true effect, the overall mixed results reported in the current literature highlight the critical need for further high-quality, well-designed randomised clinical trials.

### 6.1. Healthy Ageing Animals

There is a lack of studies examining cognitive improvements in non-diseased animal trials. This highlights a potential gap in research that warrants further exploration. When considering healthy ageing, three studies were conducted; however, two of these studies reported no significant improvement in cognitive function [38,39]. Notably, one study that utilised CoQ10 in combination with vitamin E did yield positive results, suggesting that joint supplementation between the two may hold promise in their effectiveness of enhancing spatial memory and learning in healthy ageing [40]. However, as CoQ10 administered alone did not appear to benefit cognition this study, it is unclear to what extent effects are due to the combination of CoQ10 and vitamin E or vitamin E alone.

### 6.2. Animals with Epilepsy and Alzheimer’s Disease

A single epilepsy study indicated an enhancement in cognitive function and a reduction in oxidative stress when CoQ10 was taken to reverse the damage done by antiepileptic drugs like phenytoin. This finding is significant as it suggests potential neuroprotective mechanisms when facing oxidative stress and learning due to the chronic use of an antiepileptic. Given that this was conducted on only one antiepileptic agent, further research should be conducted to determine if it is beneficial for additional antiepileptic therapies [50].

The findings from studies on AD present a promising therapeutic potential. Out of nine studies, all demonstrated improvements in cognition (as seen in Table 1 and Table 2), namely in the ability to learn [41,42,43,44,45]. These improvements were associated with reductions in histological markers of AD, lower oxidative stress levels, and increased ATP production [42,43,44]. A study demonstrating the protection of 12 hippocampal proteins, including CPLX-1 and CPLX-2, reinforces the hypothesis that targeted interventions like CoQ10 may help mitigate the progression of cognitive decline associated with AD [45].

### 6.3. Healthy Humans and Cognitive Impairment Conditions

In human studies, the results are mixed. For healthy humans, one CoQ10 study yielded no effect, while another demonstrated borderline cognitive improvement [60,61]. A recent study of healthy ageing is ongoing, with results to be released in 2025 [68]. In AD research, one study similarly found no significant impact, reflecting the complexity and variability inherent in cognitive interventions [62].

Mild cognitive impairment, a precursor to dementia, also showed no improvement in one study. However, an enhancement in cerebral vasoreactivity was noted, although its clinical relevance remains unclear given the lack of cognitive effects [63]. In the context of Parkinson’s disease, two studies produced contrasting outcomes: one study reported positive effects, while another found no significant impact, indicating the need for more comprehensive research in this area [64,65].

### 6.4. Humans with Diverse Neurological Disorders

A study on progressive supranuclear palsy yielded favourable outcomes, as did a study of CFS, which demonstrated cognitive enhancements [66,67]. These results are particularly compelling, as they may facilitate the comprehension of cognitive impairments across diverse neurological disorders.

### 6.5. CoQ10 in Cognitive Mechanisms

Potential mechanistic pathways underpinning cognitive effects (particularly effect upon inflammation and oxidative stress and histological markers associated with Alzheimer’s disease) within the literature discussed here were predominantly examined in animal studies. While there were few human trials likewise exploring potential mechanisms of effect, there is considerable evidence that elevated inflammation oxidative stress and poorer cardiovascular and cerebrovascular health inferred via reduced cerebral blood flow each predict poorer cognitive performance in humans [5,6,7,29,30,79,80]. Importantly, various systematic reviews and meta-analyses have reported that supplementation with CoQ10 is capable of lowering inflammation, reducing oxidative stress, and lowering blood pressure [20,37,81,82,83]. Regarding these potential mechanisms, our review identified improvements in oxidative stress markers in studies involving patients with AD and epilepsy, while reductions in inflammatory markers were observed exclusively in studies on AD [44,46,47,50]. Improvements in cardiovascular function were also reported in trials involving individuals with MCI (cerebral vasoreactivity) and healthy adults (cerebral blood flow), although the latter was limited by the confounding influence of multiple co-administered supplements [60,63].

### 6.6. Justification and Directions for Future Studies

In studies in animals, CoQ10 has demonstrated its potential to improve cognitive function in ageing and neurodegenerative diseases and its ability to reduce oxidative stress and improve mitochondrial function in the brain. The very few clinical trials of CoQ10 that have been conducted on the effects of CoQ10 on cognition in ageing and neurodegenerative diseases can be seen in Table 1 and Table 2.

Furthermore, as previously postulated for Parkinson’s disease the earlier a diagnosis is given, allowing for a longer treatment duration, the greater the potential benefit for patient outcomes [84]. Given the current and forecast increase in society of neurodegenerative illnesses such as AD, a gap in knowledge needs to be filled. There needs to be an influx of new clinical research into therapeutic options such CoQ10 that have previously shown beneficial results in animal studies [85].

It is important to consider that observed disparities in cognitive benefits of CoQ10 supplementation reported between animal and human studies may be due to differences in BBB permeability and/or the methods of administration and preparation of the supplements used [86]. A pertinent example of the latter are the markedly high doses of CoQ10 oftentimes administered in animal studies (see Table 1). Such doses, while potentially effective, are unlikely to be feasible, certainly not sustainable, in human samples. Though such high doses of CoQ10 in preclinical animal studies can help elucidate potential mechanisms by which CoQ10 benefits cognitive performance, perhaps animal studies in this area could also explore whether similar effects are achievable via doses of CoQ10 that are more sustainable long term and likely to be well-tolerated in humans. Future studies on the purported cognitive effects of CoQ10 should also carefully consider pertinent risk factors for cognitive decline which may be beneficially modified through CoQ10 supplementation. As discussed earlier, risk factors for cognitive decline that are amenable to CoQ10 include elevated inflammation and oxidative stress, as well as poorer cardiovascular function [5,6,29,30,79,80]. However, to our knowledge, no studies have specifically sought human populations demonstrating enhanced expression of these risk factors to subsequently explore the mechanistic pathways by which CoQ10 benefits cognition (either via inducing acute improvements in performance or, as may be more likely, slowing cognitive decline over time). Additional research in the transfer of CoQ10 through the BBB and the differences between this transfer for animals and humans needs to be performed [28]. Moreover, while several studies report positive cognitive effects of CoQ10, well-powered randomised controlled trials have also shown null results, potentially due to methodological differences or limitations in measuring relevant mechanisms of action. Thus, there remains an opportunity for well-designed randomised clinical trials to not only investigate possible cognitive benefits occurring in response to CoQ10 supplementation but to also identify viable mechanistic pathways which may underpin potential cognitive benefits.

Future research must evaluate and justify both participant quantity and assessment methodologies based on prior literature to attain disciplinary uniformity. Additionally, an array of cognitive tasks needs to be completed that allows for insight into each area of cognition individually, to provide a comprehensive overview of cognition. Further, while CoQ10 is generally regarded as a safe supplement, there have been reports of some gastrointestinal effects including abdominal pain and soft stools, Arenas-Jal et al. highlight several factors which may have explained these negative effects in the relevant studies. Moreover, the reported safe level of CoQ10 in humans is 1200 mg daily, which is well below doses typically used as well as doses previously administered in human trials (see Table 2) [87]. However, polypharmacy should be considered when examining the cognitive effects of CoQ10 as negative interactions with medications such as warfarin and antihypertensive medications which may increase the risk of negative health outcomes and potentially confound any subsequent cognitive effects [87]. The result of a recently completed registered prospective, randomised clinical trial, which satisfies most of these criteria, will soon be published [68].

## 7. Conclusions

CoQ10, a naturally occurring antioxidant essential for mitochondrial energy production, has demonstrated clinical benefits in cardiovascular health and physical performance and a role in reducing oxidative stress and increasing cerebral blood flow. These findings support the therapeutic potential of CoQ10 for improving cognitive function. Studies in animals have demonstrated the potential to improve cognitive function in ageing and neurodegenerative diseases and the ability for CoQ10 to reduce oxidative stress and improve mitochondrial function in the brain. However, clinical trials with humans have produced mixed results as to cognitive benefits in response to CoQ10 supplementation. Despite this, there is good evidence to suggest that the several mechanisms that maintain optimal cognition are positively impacted by CoQ10 therapy. To fully evaluate the benefits of CoQ10 on cognitive function in ageing and in neurodegenerative diseases, additional well designed, high quality randomised clinical trials are required. Such studies could target risk factors associated with cognitive decline that are also amenable to CoQ10 treatment (e.g., oxidative stress, inflammation, poor cardiovascular health) and examine treatment effects in a broader array of cognitive functions. Recruitment of larger study samples is also essential to ensure greater statistical power and robustness of findings.

## Figures and Tables

**Table 1:** Animal studies of CoQ10 and cognition.

Condition	Authors	Animal	N	Study Length	CoQ10 Dosage	Cognitive Assessments	Outcome
Ageing	Shetty et al. (2014) [38]	Male C57BL/6j mice	119	10 weeks	CoQ10 109 mg/kg/d; or CoQ10 109 mg/kg/d and alpha-tocopherol acetate 250 mg/kg/d	Morris water maze ○ Spatial memory and learning Discriminated avoidance test ○ Learning	No improvement in cognitive performance.
	Sumien et al. (2009) [39]	Male C57BL/6 mice	150	21 months	CoQ10 0.72 mg/g; or 2.81 mg/g	Morris water maze ○ Spatial memory and learning	CoQ10 treatment had no significant improvements in cognition. Increase in CoQ10 in the cerebral cortex with treatment.
	Mcdonald et al. (2005) [40]	Male C57BL/6JNia mice	34	14 weeks	CoQ10 123 mg/kg or CoQ10 123 mg/kg and alpha-tocopherol acetate 200 mg/kg	T-maze ○ Spatial memory and learning	Combination treatment increased avoidance responses, but CoQ10 alone did not.
Alzheimer’s Disease	Dolatabadi et al. (2012) [41]	Male Wistar rats (280–320 g)	52	21 days	CoQ10 10 mg/kg in corn oil	Passive Avoidance leaning test ○ Learning	Protected memory performance.
	Dumont et al. (2011) [42]	Tg19959 mice	12	3 months	0.4% CoQ10 in Chow	Morris water maze ○ Spatial memory and learning	Treatment showed a greater ability to learn. Histological markers of AD lower.
			27	5 Months	2.4% CoQ10 in Chow		
	Fouad (2020) [43]	Male Wistar rats (110–130 g)	30	40 days	CoQ10 10 mg/kg body; or Omega 3 500 mg/kg and CoQ10 10 mg/kg	Rewarded T-maze test ○ Spatial memory and learning	Faster maze completions. Near normal brain histology.
	Ishrat et al. (2006) [44]	Male Wistar rats (480–520 g)	40	3 weeks	CoQ10 10 mg/kg	Morris water maze ○ Spatial memory and learning	Enhanced learning. Reductions in oxidative stress and elevations in ATP
	Ying et al. (2017) [45]	Triple transgenic mice of AD (3xTg-AD); and Wild-type mice (strain B6129SF2/J)	44 ± 4	3 months	CoQ10 800 mg/kg	Morris water maze ○ Spatial memory and learning	Increase in memory. Hippocampal proteins protected cognitive function.
	Attia et al. (2020) [46]	Rats	64	60 days	CoQ10 10 mg/kg, per os PO in corn oil; CoQ10 10 mg/kg, PO in corn oil and alumuminium chloride coadministered with biotin 2 mg/kg, IP, biotin and CoQ10 at the same doses.	Morris water maze ○ Spatial memory and learning	Alleviated cognitive impairments. Reduced neuroinflammation.
	Singh et al. (2015) [47]	Male Sprague-Dawley rats (180–200 g)	120	21 days	CoQ10 20 and 40 mg/kg, galantamine 2 mg/kg, minocycline 50 and 100 mg/kg and their combinations	Morris Water maze ○ Spatial memory and learning	Improved memory learning. Markers of reduced neuroinflammation. Restored mitochondrial function.
	Sheykhhasan et al. (2022) [48]	Wistar rats (250–300 g)	40	5 days	CoQ10	Morris Water maze ○ Spatial memory and learning Passive avoidance task ○ Learning	Improved memory. Histological marker of AD lower.
	Muthukumaran et al. (2018) [49]	Male Transgenic male mice; Male C57BL/6 wildtype mice	23	18 months	CoQ10 50 µg/mL	Y-maze ○ Spatial Working Memory	Improved long-term memory. Histological markers of AD lower.
Epilepsy	Tawfik (2011) [50]	Male Wistar rats (200–250 g)	40	2 weeks	CoQ10 10 mg/kg	Passive avoidance task ○ Learning	Improved Learning. Reductions in oxidative stress.
Parkinson’s Disease	Abu-Elfotuh et al. (2022) [51]	Male Sprague Dawley rats (300–320 g)	84	5 weeks	CoQ10 200 mg/kg PO; CoQ10 200 mg/kg, Sesamol 15 mg/kg, Thymol 30 mg/kg, and Wheat Grass 100 mg/kg.	Y-maze ○ Spatial Working Memory	Improved Spatial Memory. Reduced Oxidative Stress and neuroinflammation. Apoptotic pathways modulated.

**Table 2:** Human RCTs that assess the effects of CoQ10 on Cognition.

Condition	Authors	N	Treatment Duration	CoQ10 Dosage	Cognitive Assessments and Cognitive Domains	Outcome
Healthy Humans	Kennedy et al. (2016) [60]	97	8 weeks	CoQ10 4.5 mg/d, and Supradyn 1RDA	Serial Subtractions ○ Working memory and attention 3-backTask ○ Working memory Stroop Task ○ Global inhibition and processing speed Kay Tapping Control Task ○ Global cognitive function	No significant cognitive difference between groups. CoQ10 and 1RDA treatment significantly improved cerebral blood flow.
	Kinoshita et al. (2021) [61]	90	34 weeks	Ubiquinol 100 mg/d	Atamano Kenkou Chekku ○ Global cognitive function Trail Making Test ○ Visual attention and processing speed Digit Symbol Substitution test ○ Executive function	Borderline evidence of Improved cognitive function.
Mild to Moderate Alzheimer Disease	Galasko et al. (2012) [62]	78	16 weeks	CoQ10 400 mg 3 times/d	MMSE ○ Global cognitive function	No significant results for cognitive measure.
Mild Cognitive Impairment	García-Carpintero (2021) [63]	69	12 months	Ubiquinol 200 mg/d	Trail Making Test ○ Visual attention and processing speed Spain-Complutense Verbal Learning Test ○ Verbal learning and Memory Digit Span ○ Short-term memory and working memory Verbal Abstract Reasoning Test ○ Abstract reasoning Visuospatial span Test ○ Visuospatial working memory Animal list generation ○ Verbal recall and semantic fluency Boston Naming Test ○ Language Token Test ○ Language Rey-Osterrieth Complex Figure Test ○ Visuo-constructional ability and visual memory	No statistically significant results for cognitive measures. Ubiquinol significantly improved cerebral vasoreactivity.
Parkinson’s Disease	Beal et al. (2014) [64]	600	16 months	CoQ10 1200 mg/d or CoQ10 2400 mg/d, each with 1200 IU/d of vitamin E	Symbol Digit Score ○ Sustained attention and working memory	No significant cognitive difference between groups.
	Li et al. (2015) [65]	75	18 months	CoQ10 100 mg t.i.d and Creatine 5 g b.i.d	MoCA ○ Global cognitive function	Improved MoCA scores after 12 and 18 months of treatment.
Progressive Supranuclear Palsy	Stamelou et al. (2008) [66]	21	6 weeks ± 4 days	CoQ10 5 mg/kg/d	MMSE ○ Global cognitive function FAB ○ Executive Function	Improved FAB scores with treatment.
Chronic Fatigue Syndrome	Fukuda et al. (2016) [67]	20 + 43	8 weeks + 12 weeks	Ubiquinol 150 mg/d	Arithmetic Task ○ Executive function	Improved arithmetic task performance with Ubiquinol treatment.

Abbreviations: MMSE, Mini Mental State Exam; MoCA, Montreal Cognitive Assessment; FAB, Frontal Assessment Battery.

## Data Availability

No new data were created or analysed in this study. Data sharing is not applicable to this article.
